# Microstructure, Mechanical and Wear Behaviour of Deep Cryogenically Treated EN 52 Silchrome Valve Steel

**DOI:** 10.3390/ma15165484

**Published:** 2022-08-10

**Authors:** Iyyanar Saranraj, Sudalaimuthu Ganesan, Lenka Čepová, Muniyandy Elangovan, Libor Beránek

**Affiliations:** 1 Department of Mechanical Engineering, Vel Tech Rangarajan Dr. Sagunthala R&D Institute of Science and Technology, Avadi 600062, India; 2 Department of Machining, Assembly and Engineering Metrology, Faculty of Mechanical Engineering, VSB-Technical University of Ostrava, 70800 Ostrava, Czech Republic; 3 Department of R&D, Bond Marine Consultancy, London EC1V 2NX, UK; 4 Department of Machining, Process Planning and Metrology, Faculty of Mechanical Engineering, Czech Technical University in Prague, 16600 Prague, Czech Republic

**Keywords:** EN 52 Silchrome steel, deep cryogenic treatments, mechanical properties, wear surface morphology

## Abstract

This study has compared the performance of cryogenically processed EN 52 Silchrome valve steel with untreated material. After completing the standard heat treatment process, EN 52 steel material specimens are subjected to a deep cryogenic process with varying soaking temperatures. The parameters of the deep cryogenic procedure were changed to find the best wear qualities. The key features of valve steel, such as microstructure, mechanical, and wear behaviour are evaluated by conducting a test study. The evolution of wear mechanisms after enhancing qualities of EN 52 steel is studied using scanning electron microscopy. The mechanical and wear behaviour improved due to factors such as fine carbide precipitation, conversion of residual austenite, and carbide refining formed after cryogenic treatment. With a maximum reduction in wear rate of up to 45%, the deep cryogenic treatment of EN 52 steel with a soaking temperature of −140 °C was the ideal parameter. All three cryo-treated samples had better properties than the untreated EN 52 valve steel.

## 1. Introduction

DCT (Deep Cryogenic Treatment) improves a material’s mechanical and physical properties by cooling it to low temperatures. The DCT process parameters are crucial in determining the material’s characteristics. Engine components such as valves have a critical role in deciding the engine’s life and efficiency in automotive applications. EN 52 steel is the most commonly utilized material for intake and exhaust valve stems in I.C. engines. Multiple treatments are carried out as research activities to improve the life and performance of engine components. Cryogenic treatment is one such method for improving performance. In recent years, low-temperature treatments have positively impacted steel performance. Cryogenic treatments are a cost-effective and long-lasting method that improves the entire component. It is an additional method used along with the traditional heat treatment. The samples are rapidly cooled to cryogenic temperatures, held for an extended period, and then heated to ambient temperature and tempered to improve ductility. Significant effects include improvements in mechanical characteristics and modifications in the microstructure. Cryogenic treatment is more efficient than traditional heat treatment. Cryogenic treatments are recommended to enhance the durability of automobile components. As a result of cryogenic treatments, steel microstructures alter significantly. Their element composition and hardening process determine the mechanical characteristics of metals. The mechanical qualities of valve steel are tensile strength, yield strength, hardness, elongation, and wear behaviour. High tensile strength and wear tolerance are required for engine valves.

Many researchers have investigated the impact of cryogenic treatment on engine components and other automobile parts to improve performance. Following, they have reported the benefits of cryo-treating steel as a restoring method. Deep cryogenic treatments (DCT) are performed at temperatures ranging from −125 to −196 °C, whereas standard cryogenic treatments (SCT) are performed at temperatures ranging from −60 to −80 °C. Beyond the SCT process, DCT increases certain features [1]. The three essential elements in cryogenic metal treatments are the cooling rate, soaking time, and quenching temperature.

The morphology and mechanical characteristics of heat-treated 0.4C-Si-Mn-Cr steel were investigated by Zhang et al. (2020). After heat treatment, the steel’s hardness and impact toughness were both strengthened [2]. The cryogenic treatment causes fine and uniformly dispersed carbides to precipitate in valve steel, which improves its mechanical qualities. Hardness, wear resistance, and tensile strength will benefit from the adjusted DCT process settings [3]. The complete transition of austenite to martensite structure and the formation of fine carbides due to cryogenic processing of En 31 steel have improved its wear resistance and hardness [4]. En 19 steel was applied to DCT, SCT, and CHT processes, and the wear resistance of all samples was compared by Senthil Kumar et al. (2011). DCT samples of En 19 steel showed the most improvement in wear [5].

On the other hand, Huang et al. (2003) pointed out that fine carbide precipitation and the transition of residual austenite into martensite help to strengthen wear resistance [6]. The formation of tiny carbide particles improves hardness and the wear resistance for SCT and DCT samples, according to microstructure investigations of En 31 steel [7]. The development of fine carbides and their homogeneous distribution has enhanced wear after cryogenic treatment of valve steel materials. As a result, cryo-treated valves will last longer and perform better [8].

The effects of DCT on the mechanical and microstructure of the medium carbon steel EN 52CrMoV4 were investigated by Ozden et al. (2020). The steel’s mechanical characteristics have been improved by producing uniformly dispersed fine carbides in the microstructure [9]. Abbas 2019 Dissolving of austenite, homogeneous carbide distribution, and precipitation of fine secondary carbides contribute to increased hardness in most steels [10]. Das et al. (2009) evaluated the wear parameters of D2 steel after cryo treatment to determine the optimal soaking period for maximum wear resistance. The samples treated for 36 h had the most robust wear resistance [11]. The effect of DCT on 7Mn steel was investigated by Sun et al. (2019) to determine its mechanical behaviour. The material has significantly benefited from the DCT treatment combined with inter-critical annealing [12].

The wear and tensile tests of cryogenic treated and tempered H13 tool steel were studied by Cicek et al. (2015). They discovered that DCTT samples were more resistant to wear in all situations [13]. Vimal et al. (2008) have used the DCT technique to investigate the tribological behaviour of En 31 steel. They discovered that wear could be decreased by up to 75% depending on the operating conditions [4]. Sugavaneswaran et al. (2019) have evaluated the effect of cryo treatment on the wear behaviour of 316L stainless steel. The coefficient of friction values has been reduced in cryo-treated samples compared to parent metal [14]. Vahdat et al. (2014) tested the DCT technique on 1.2542 tool steel to determine the density of secondary carbides. They discovered that increasing the soaking period resulted in a higher volume of secondary carbides [15]. Kumar et al. (2018) examined the M2 tool steel material’s behaviour to varying DCT holding times to determine its wear parameters. Compared to 12 and 36 h, the 24-h holding duration yielded more excellent wear resistance [16]. Kaya et al. (2020) have tested the cryogenic treated 38MNVS6 medium carbon steel to find its wear performance. The outcomes demonstrated that harder and more wear-resistant tribological qualities were improved by applying longer soaking cryogenic treatment periods [17]. Amini K et al. (2016) have investigated the deep cryogenic treated 1.3255 tool steel subjected to wear test. The improvement in hardness and wear resistance following DCT was associated with a drop in retained austenite level, the precipitation of fine carbides, and a large proportion of the carbides [18]. Amini et al. (2016) have examined the effect of DCT on the corrosive-wear behaviour of the 16MnCr5 steel. The decrease in dissolved chromium atoms in the microstructure causes a rise in the wear rate of DCT specimens [19]. The amount of chromium plays a major role in the wear behaviour of the steel. Kandeva et al. (2021) have investigated the influence of chromium on the abrasive wear of Ni-Cr-B-Si coatings applied by a supersonic flame jet. It has been shown that upon increasing the concentration of chromium, the wear rate decreases non-linearly, reaching minimal values at 16% Cr [20].

It is well established that cryogenic treatment is used on various materials and under various conditions. Adjustment in cryogenic treatment parameters and levels will result in a wide range of property enhancements. Steel should be treated with distinct cryogenic treatment parameters to determine the behaviour under ideal circumstances. Cryogenic treatment of various valve steels has increased its characteristics, as we know from previous research. As a result, it is recommended that the specific cryogenic treatment conditions for EN 52 valve steel be determined.

According to the literature review, engine valve wear is among the essential elements affecting valve lifespan, and cryogenic processing can improve valve steel wear resistance [21,22,23,24,25]. The current study compares the mechanical properties and wear resistance enhancement in the sample employing DCT and a wear monitor, as well as a surface morphology analysis, to determine the impacts of cryogenic treatment on EN 52 valve steel. The valve wear is a complex issue, and we have tried to increase the wear resistance by applying cryogenic treatment, which helps to enhance the hardness and precipitate fine carbides. Hence an attempt has been made to study the effect of DCT parameters on the mechanical and wear behaviour of EN 52 valve steel.

## 2. Material and Experimental Process

For this research, EN 52 silicon chromium valve steel was chosen. Optical emission spectroscopy (OES) was used to confirm the chemical composition of the material (see Table 1). The specimens for the wear tests were machined according to the specifications. The produced samples were divided into three groups and treated in three distinct processing methods.

### 2.1. DCT Method

**Figure 1:**
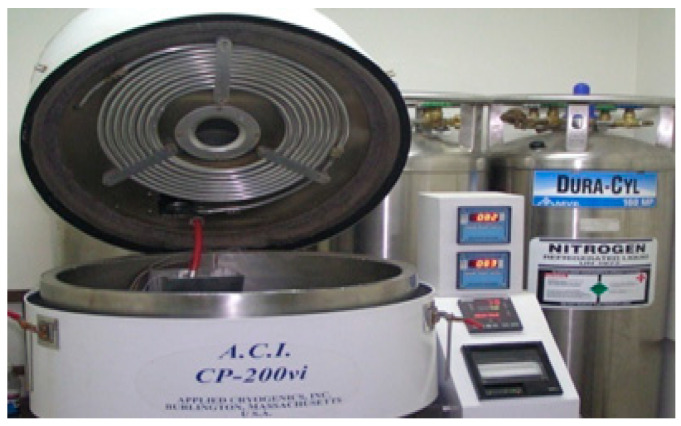
Cryogenic treatment processor equipment [26].

The factor evaluated in DCT of EN 52 steel is the soaking temperature, which has three different ranges, as indicated in Table 2. The heating rate was set at 1 °C/min to avoid thermal cracking. The specimens were hardened at 975 °C for 1 h and then oil-quenched. The steels are then processed in a cryogenic device according to the specifications chosen. The cryogenic processor in Figure 1 has a fully insulated compartment and liquefied nitrogen kept in a tank utilized as the operating fluid. The PID controller regulates the flow (liquefied nitrogen) depending on the chamber’s temperature.

A programmable temperature controller was used to control cryogenic treatment variables such as soaking temperature, soaking time, and tempering temperature. The details of the DCT process are captured and stored using a data platform. The hardened samples were cooled at a constant rate, held at a specific temperature for an extended time, and then gradually warmed to their original state. The specimens were then tempered for 1 h.

### 2.2. Mechanical Properties Test

The tensile strength tests were performed on DCT EN 52 steel samples following ASTM norms. The tensile test was performed on an ‘FIE’ Electronic Universal Testing Machine (UTS-100), with round bar specimens and ASTM E8-04 tensile specimen parameters. The hardness measurement was performed under the ASTM standard E 92-82 (standard testing procedure for Vickers hardness of metals). The Vickers hardness test was performed so that each specimen obtained three indentations. The load chosen for the test was 30 Kgf, which was applied for 10 s (dwell time).

### 2.3. Wear Test

The wear test was performed using pin on disc wear test equipment (DUCOM TR 20 LE model—DUCOM Instruments Pvt. Ltd., Bangalore, India) on samples subjected to the three separate parameters of the DCT process, according to ASTM standard G-133 (17). The wear rate of the valve steel specimens during rotation was assessed to determine the materials’ wear resistance. The wear test employed specimens with a diameter of 6 mm and a length of 10 mm. The material samples are cleaned with acetone to remove any impurities before being cleaned for 10 min with an ultrasonic cleaner.

The response parameters of specific wear rate (Ws) and coefficient of friction (µ) were used to assess the wear behaviour [27,28]. The applied load characteristics are adjusted according to the values chosen. The specimens are thoroughly polished with emery paper and then cleaned with kerosene. Before and after the test, the weights of each sample were recorded, and they were used to estimate wear loss and wear rate (mg/m × 10^−4^).

### 2.4. Optical Microscope

The sample EN 52 steel specimen was subjected to surface preparation for optical microscopy by the etching process. The etching solution used for the process contained 1- part Nitric acid and 3-parts distilled water by immersion. The microscopy was performed with the optical microscope with a magnification of 1000×. The microstructure investigation was performed with LEICA optical microscope to study the behaviour of DCT on EN 52 steel.

### 2.5. Wear Studies Using SEM

SEM examination allows for the characterization of the fracture surfaces of the specimen following the wear test at high magnifications and the observation of surface characteristics [29,30]. At 1000× and 5000× magnification, the fracture surfaces are visible clearly, which aids in identifying the underlying process. A Thermo scientific Apreo model HRSEM (5–300,000×) was used for the examinations.

## 3. Results and Discussion

### 3.1. Mechanical Properties

Based on previous literature, the modification in soaking temperature had a non-linear effect on the mechanical characteristics of EN 52 steel, which was considered for this study. The cryo-treated EN 52 steel with higher soaking periods resulted in better tensile and wear behaviour. Hence, our study selected a constant value of 24 h soaking period.

The tensile test results are reported in Table 3 for the DCT treated EN 52 valve steel samples prepared with varying soaking temperatures. The non-cryo-treated EN 52 valve steel specimen has UTS of 1063 MPa, and an enhanced tensile strength of 1185 MPa was achieved for the DCT3 sample after cryo treatment. When the findings are compared, the DCT3 samples improve better than the other specimens. All the DCT sample shows better strength compared to non-DCT samples. Compared to non-treated EN 52 steel, the treated material shows a maximum increase of 122 MPa for UTS. The DCT1 samples had a 28 MPa higher value in tensile strength than the DCT2 samples. The transition of the residual austenite into martensite and the formation of fine secondary carbides (SC) are attributable to the increase in UTS, as evidenced by the microstructure study conducted earlier by Jaswin et al. [2,31].

According to the Vickers hardness test results, the EN 52 valve steel’s hardness has slightly improved due to the cryogenic treatment. Compared to the DCT, the Vickers hardness has increased by 43 HV, as shown in Table 3. The cryogenic process is more efficient at reducing austenite and can precipitate a greater quantity of fine secondary carbides, enhancing the impact of dispersion strengthening. Both of the preceding reasons are advantageous to increasing hardness. Furthermore, as is well known, decreasing grain size improves hardness.

### 3.2. Wear Behaviour

**Figure 2:**
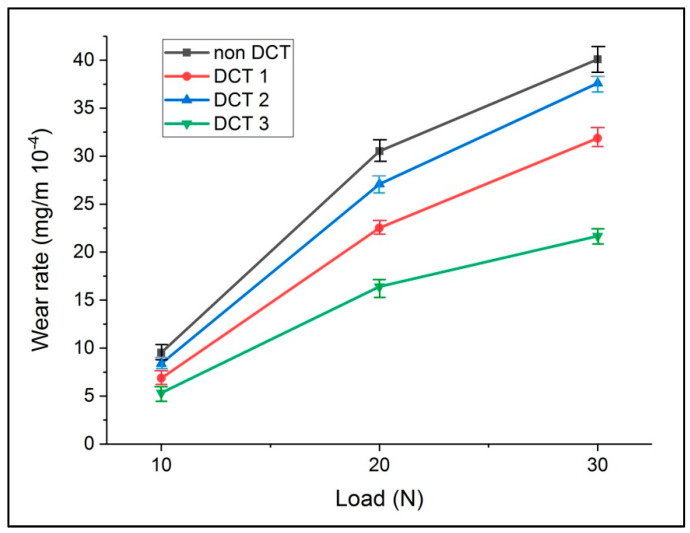
Wear rate data with varying loads of all four samples.

The effects of different loads on the cryogenically treated EN 52 valve steel samples were investigated. Figure 2 shows the enhancement in wear resistance of cryogenically processed specimens as a function of different treatment parameters. For the EN 52 steel material, the improvement in wear resistance of the DCT specimen was significant at a higher load. When compared to non-cryo-treated specimen, the wear resistance of EN 52 steel valve steels was strengthened by 20.3% owing to DCT1 and 45.8% due to DCT3 with a higher load condition of 30 N. DCT improved wear resistance by 20.5, 6, and 45.8% for DCT1, DCT2 and DCT3 specimens, respectively, as compared to non-cryo-treated samples. The DCT specimens showed a significant increase in wear resistance at all load conditions. The increase in wear resistance observed at maximum load implies that hot hardness has improved.

Figure 2 shows the wear rate for the treated EN 52 steel specimens for varied loads of 10, 20, and 30 N. According to the findings, the wear rate for the DCT specimens was lower than the non-cryo-treated specimens at all loads. Analyzing Figure 2, it is clear that wear increased almost proportionally to the applied load and frequencies in all the treatments.

According to the test results, the DCT specimens demonstrated a strong wear resistance (minimal wear weight loss) in all situations. In addition, it was discovered that the EN 52 steel had a wear rate of 31.8 × 10^−4^ mg/m, 37.6 × 10^−4^ mg/m, and 21.6 × 10^−4^ mg/m in the DCT (1, 2, and 3) specimens, respectively when analyzing the wear behaviour in the complex condition of the test (load 30 N). Hence the DCT can be used to strengthen wear resistance.

All three cryogenically treated specimens outperformed the non-cryogenically treated specimens on wear resistance. Three significant elements must be considered for enhancing the wear resistance after cryogenic treatment. The materials resist wearing during the wear test due to the precipitation of finer carbides that spread uniformly and residual austenite transformation into martensite and matrix refinement due to increased martensite transformation.

**Figure 3:**
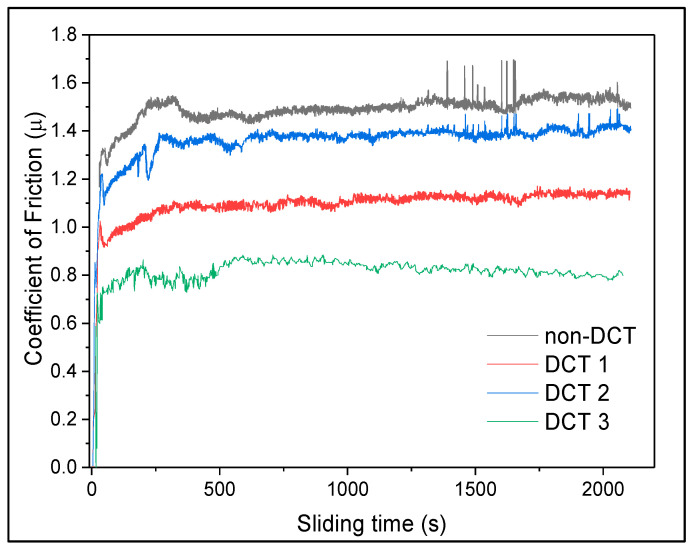
CoF data with 30 N load on all 4 samples.

Figure 3 shows the coefficient of friction (CoF) for various material situations. The CoF of the DCT material is substantially lower than that of untreated materials. Other researchers have also suggested that the surface of modified DCT material produced by intense plastic deformation could reduce CoF. The surfaces of the DCT-modified material will reduce CoF during severe plastic deformation. Furthermore, the DCT soaking temperature impacts the CoF curve’s value. Material treated at −140 °C soaking temperature had a lower CoF than material treated at the other two temperatures. With a load of 30 N, the CoF values have varied. It can also be noted that the CoF variation was more severe in the steady-state period of the testing for the DCT2 sample, which could be due to the uneven distribution of fine carbides precipitated during DCT.

### 3.3. Microstructure Studies

**Figure 4:**
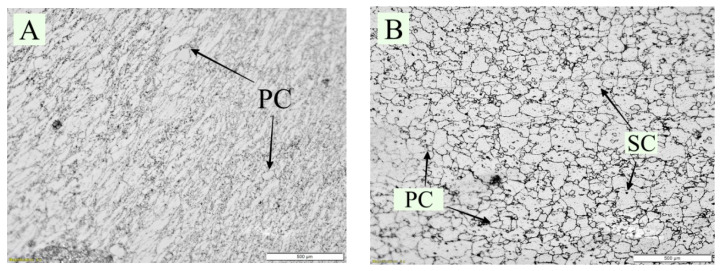

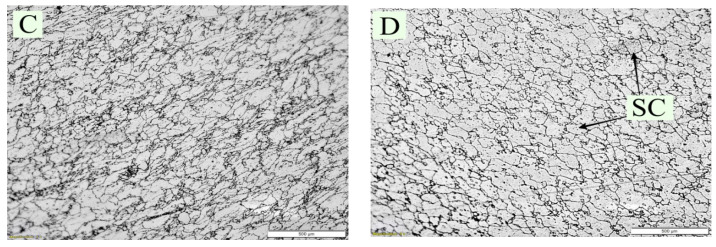
Microstructure of EN 52 steel from (**A**) non-DCT (**B**) DCT1 (**C**) DCT2 and (**D**) DCT3 samples (PC-primary carbides, SC-secondary carbides).

**Figure 5:**
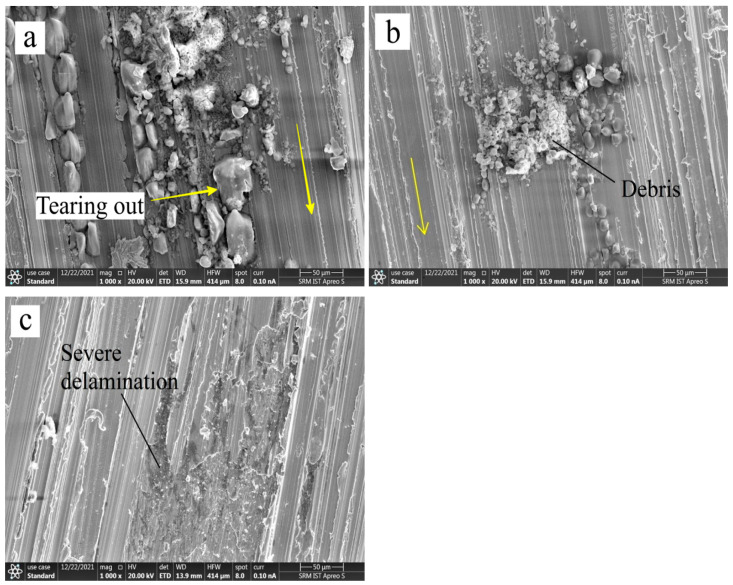
Wear morphology of EN 52 steel without DCT showing (**a**) tearing out (**b**) debris (**c**) severe wear and deformation.

Microstructural analysis was performed to investigate the modifications that affect the improvement in the properties of the EN 52 valve steel material following DCT. Figure 4 and Figure 5 depict the microstructure of the EN 52 DCT samples. The structure and particle size of the carbides seen in the micrograph are more related; however, more fine carbides are distributed on the martensitic matrices in the DCT3 samples than in the non-cryo-treated samples. Carbide refinement in huge quantities is also noticeable in the DCT3 sample. The DCT samples’ microstructure in Figure 4B–D indicates a significant rise in carbide precipitation. In the cryo-treated specimens, finer chromium carbides precipitated in the martensitic matrix. These were also in charge of the improved characteristics of the cryo-treated EN 52 valve steel. In the non-DCT sample (Figure 4A), there are more large-sized carbides than in the DCT samples. When the microstructures of the DCT samples were compared, the DCT3 sample had a more significant proportion of fine carbides precipitated.

### 3.4. Wear Surface Morphology Using SEM

**Figure 6:**
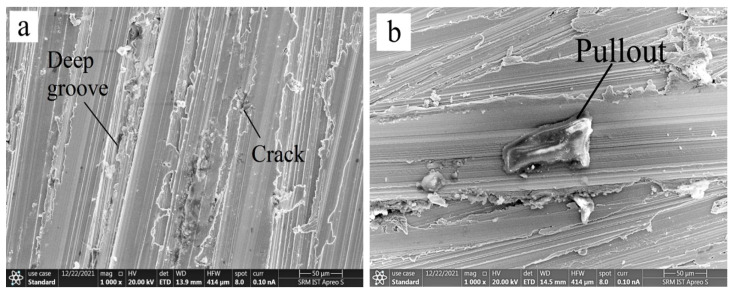
Wear morphology of DCT2 sample showing (**a**) cracks and (**b**) deep grooves.

**Figure 7:**
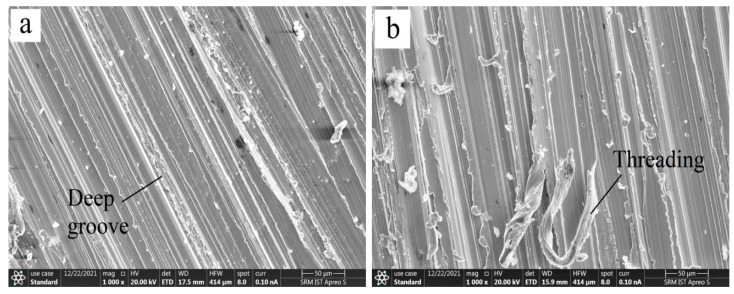
Wear morphology of DCT 1 sample showing (**a**) deep grooves and (**b**) thread-like pullout.

**Figure 8:**
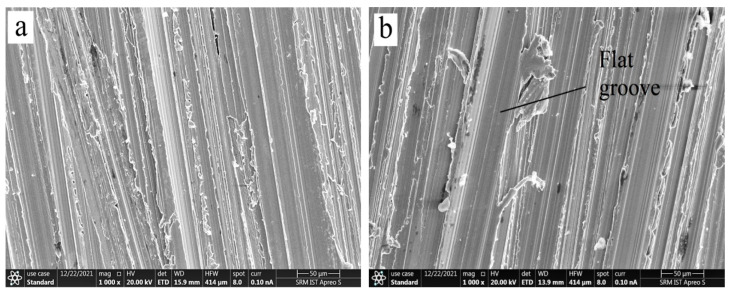
Wear morphology of DCT 3 sample showing (**a**) limited grooves and (**b**) flat grooves with reduced wear loss.

SEM analysis was used to examine the worn path of EN 52 steel and cryo-treated EN 52 steel to recognize the wear mechanism better. SEM was used to analyze the worn regions of the samples after conducting the wear tests. Figure 5, Figure 6, Figure 7 and Figure 8 show SEM pictures of the wear scar area, wear traces, and the comparative wear surface results.

The distortion in the untreated EN 52 steel sample was severe, as shown in Figure 5. However, the deformation in the cryogenically treated sample was considerably decreased (see Figure 6). The plastic deformation was shown in SEM micrographs (Figure 5), with flow lines along sliding directions marked in yellow arrow marks and several micro-cracks indicating that the surface had experienced significant contact stresses during sliding.

According to the SEM findings, carbide particles begin to separate from the sintered disc material, i.e., hardened steel, during the initial period of wear testing, resulting in a quick increase in the friction coefficient. As the particle body and the specimen were made of the same material and ductile, significant distortion and plastic flow were seen in the wear track, both features of adhesive wear [14]. Wear debris was visibly embedded in large carbide particles found in the wear track of the untreated EN 52 steel sample. However, it was not visible in the cryogenically treated sample wear track due to the precipitation of finer carbides. Due to this event, the wear track had a differentiated surface morphology before and after the DCT of the EN 52 sample.

The loose wear debris increases the effective contact area between the disc and the specimen, resulting in increased contact pressure and more severe wear in the untreated sample than in the DCT sample [14]. So there is a considerable increase in COF in the transformation stage of the EN 52 sample that has not been cryo-treated. The pin sliding against the disc causes a significant temperature rise in the sliding surface, resulting in the surface’s preferred oxidation.

The wear surface of the untreated EN 52 sample reveals uneven patterns of the wear grooves along the track lines and apparent indications of abrasive indentation in pores. The wear track of the cryogenic treated EN 52 sample is comparatively smooth and shallow compared to the wear track of the untreated EN 52 sample, which has big peaks and valleys (Figure 5), showing that the cryogenic treated EN 52 sample has minor wear. These findings determined that material removal is higher during wear testing in the EN 52 sample before cryogenic treatment.

Wear debris is produced mainly by the relative motion of contact surfaces under load. Due to continuous sliding with stresses, much debris is ejected from the contacting interface, resulting in material wear loss, but other debris is maintained between the rubbing surfaces. As moving particles plow the surfaces to create grooves, this debris persists and agglomerates in the contact zones. As a result, as shown in Figure 8, minor grooves can be found in the worn portions of all materials. Abrasive wear during sliding is responsible for forming grooves and slip areas [14]. As the sliding progressed, some of the debris particles that remained in the interface became severely compacted, forming a localized tribo-oxide layer overlying a portion of the worn surfaces.

The debris accumulation activity of the three specimen categories differed. The tribological surface on the untreated specimen has been noticed to be more aggregated than those on the cryogenically treated specimen, as shown in Figure 5. This behaviour might be related to debris with lower hardness spalled from the untreated materials being simpler to densify in the interaction. Owing to the greater hardness and compressive residual stress of the surface and subsurface of the cryo-treated specimen, they have improved comparative wear resistance, as shown in Figure 8. Micro-scale debris from the treated materials is thought to preserve more refined grains and higher toughness. As a result, combining such material to form localized tribolayers is more challenging. The surfaces were delaminated as the counter face (the steel disc) was pressed and slid. Figure 6 and Figure 7 show that the broken layers and debris agglomerated along the margin of the worn area. As a result, adhesion and delamination are found as wear processes in EN 52 cryo-treated material.

## 4. Conclusions

The main findings are derived from the above-mentioned experimental findings and discussions.

- The study reveals that DCT increased the wear resistance of EN 52 valve steel. The cryogenic treatment significantly improved wear resistance, with 45% less wear for DCT 3 sample.
- The DCT increases the UTS of EN 52 valve steel. The increase in wear resistance observed at maximum load suggests that the materials’ hot hardness has improved.
- According to the wear surface morphology analysis, the DCT produces the precipitation of smaller carbides with a more significant volume proportion and homogeneous dispersion throughout the structure of EN 52 steel.
- The precipitation of ultrafine carbides by decomposing martensite was a significant element in enhancing the wear resistance of cryo-treated valve steel materials. DCT can be used to improve wear resistance, according to this study.
- According to the results of the experiments, cryogenic treatment can reduce valve train wear in engines, resulting in improved fuel efficiency and performance.

## Figures and Tables

**Table 1:** Elemental composition of EN 52 steel.

Material	C	Cr	Si	Mn	P	S	Ni
EN 52	0.45	8.6	3.3	0.6	0.04	0.03	0.4

**Table 2:** Process parameters used in DCT.

Samples	Description	Soaking Temperature (°C)
1	Non-treated	--
2	DCT1	−190
3	DCT2	−170
4	DCT3	−140

**Table 3:** Mechanical properties of EN 52 steel samples.

Sample Number	Soaking Temp. (°C)	Soaking Period (Hours)	Tempering Temp. (°C)	Tensile Strength (MPa)	Hardness (HV)
Non-DCT	-	-	-	1063	303
DCT 1	−190	24	650	1168	334
DCT 2	−170			1140	323
DCT 3	−140			1185	346

## Data Availability

The data presented in this study are available through email upon request to the corresponding author.
